# Kazakh banknote image dataset

**DOI:** 10.1016/j.dib.2026.112634

**Published:** 2026-02-26

**Authors:** Ualikhan Sadyk, Makhambet Yerzhan, Cemil Turan, Haohan Wang

**Affiliations:** a Department of Computer Science, SDU University, Abylaikhan St. 1/1, Kaskelen, Kazakhstan; b Faculty of Computing & Data Sciences (CDS), Boston University, 665 Commonwealth Ave, Boston, MA 02215, USA; c School of Information Sciences, University of Illinois at Urbana Champaign, 501 E. Daniel St. MC-493 Champaign, IL 61820-6211, USA

**Keywords:** Central Asian currency, Banknote recognition, Currency detection, Machine Learning

## Abstract

This paper presents an image dataset of Kazakh banknotes collected for research in computer vision and pattern recognition. The dataset contains photographs of banknotes from seven categories corresponding to the denominations 500, 1000, 2000, 5000, 10,000, 20,000 Kazakhstani tenge, as well as a mixed category containing multiple denominations within a single image. Each denomination folder includes at least 100 images, while the mixed category contains over 1000 images. Images were captured against a wide range of backgrounds and under varying lighting conditions to reflect realistic usage scenarios and environmental diversity.

The 5000 tenge denomination is further divided into three subsets that distinguish between old and new banknote designs and between single-note and multiple-note configurations. Most images contain multiple banknotes of the same denomination, except for one subset explicitly designed to include a single banknote per image. All files follow a consistent numeric naming convention.

The dataset is intended to support tasks such as banknote classification, object detection, and robustness analysis of deep learning models under background and illumination variation. It may also be reused for transfer learning, dataset bias studies, and comparative evaluations across currency recognition systems.

Specifications Table

**Table:** 

Subject	Computer Science
Specific subject area	Computer vision; currency recognition; image-based object classification and detection
Type of data	Digital color images (RGB) of physical objects (banknotes), organized into denomination-based folders and subfolders
Data collection	The data were collected by photographing Kazakhstani banknotes under varied real-world conditions. Images were captured against diverse backgrounds and with different lighting setups to introduce variability in scene appearance. Most images contain multiple banknotes of the same denomination arranged in different configurations, while a specific subset contains a single banknote per image. The 5000 tenge denomination was intentionally collected in separate subsets to distinguish between old and new design series. All images were manually reviewed, organized by denomination, and stored using a consistent numeric file-naming convention. The data were collected exclusively for research purposes and do not contain personal or sensitive information.
Data source location	SDU University Abylaikhan 1/1, Kaskelen, Kazakhstan
Data accessibility	Repository name: Kazakh Banknote Image Dataset Data identification number: DOI:10.17632/gyw87k93yb.1 Direct URL to data: https://data.mendeley.com/datasets/gyw87k93yb/1
Related research article	Sadyk, U., Yerzhan, M., Baimukashev, R., & Turan, C. (2024). Comparative analysis of machine learning approaches in Kazakh banknote classification. Bulletin of Electrical Engineering and Informatics, 13(5), 3552–3567.

## 1 Value of the Data

• The dataset provides image data of Kazakhstani banknotes, a currency that remains underrepresented in publicly available computer vision resources.
• In contrast to the existing Kazakh banknote dataset (KZ-DB) reported in *Data in Brief*, where each image contains a single banknote per frame, the present dataset explicitly includes multi-banknote scenes and a mixed-denomination (“Mix”) category. This design extends the scope from single-object recognition to more realistic scenes involving clutter and multiple instances [1].
• By covering multiple denominations and including design variation for the 5000 tenge banknote (old and new series), the dataset captures visually meaningful changes encountered in circulation. The 5000 tenge images are further organized into subsets representing single-banknote and multiple-banknote compositions, enabling controlled comparisons between single-instance and multi-instance settings within the same denomination.
• The dataset is directly reusable for supervised image classification tasks (denomination recognition at the image level) and for evaluating preprocessing, data augmentation, and feature extraction strategies under background and illumination variability. Reproducibility is supported through a file inventory (manifest.csv with SHA-256 checksums) and predefined train/validation/test split lists (splits/).
  Although the dataset does not include bounding boxes, segmentation masks, or per-instance labels, it remains valuable for detection-oriented and multi-object research workflows. In particular, it can be used for generating additional annotations, studying annotation protocols and labeling strategies (especially in mixed-denomination settings), and evaluating weakly supervised, semi-automatic, or human-in-the-loop annotation pipelines.
• The “Mix” category provides a natural testbed for analyzing model behavior in mixed-content frames where multiple denominations co-occur. This subset is therefore best suited for robustness analysis, weakly supervised learning, mixed-scene screening tasks, or downstream annotation workflows, rather than immediate fully supervised denomination labeling.
• Finally, the dataset can serve as a target or auxiliary resource for comparative studies, transfer learning, and robustness analyses, enabling complementary evaluation alongside existing single-banknote datasets.

## 2 Background

The dataset was compiled for research on image-based currency recognition under realistic conditions. Many publicly available currency datasets are dominated by a small set of widely circulated currencies and frequently emphasize controlled acquisition or single-object images, which limits their applicability to scenarios involving clutter, multiple instances, or mixed content [[2], [3], [4]]. For Kazakhstani tenge specifically, an existing KZ-DB provides useful coverage but focuses on one banknote per image, which constrains studies that require multi-instance frames or mixed-denomination composition [1,5].

The motivation for collecting the present dataset was therefore two-fold: (i) to expand coverage of Kazakhstani banknotes across multiple denominations and (ii) to include scene configurations that more closely reflect real use, namely, multiple banknotes in a single frame and mixed-denomination images [6]. Particular attention was also given to distinguishing old vs. new design series for the 5000 tenge banknote and to separating single-banknote versus multiple-banknote compositions for this denomination, reflecting differences in both visual appearance and scene complexity encountered in circulation.

The data article documents the dataset organization, curation, and reproducibility supports (inventory and predefined splits) to facilitate consistent reuse. Given the absence of pixel-level annotations, the dataset is positioned primarily for denomination recognition and methodological evaluation at the image level, while also enabling downstream extension to instance-level tasks through additional annotation efforts and annotation-generation workflows, especially in the mixed-denomination setting [7].

## 3 Data Description

The dataset is distributed as a directory-based image repository in which the directory hierarchy encodes the class labels. At the top level, each folder corresponds to a single Kazakhstani banknote denomination, with one additional folder reserved for mixed-denomination scenes. All samples are color photographs stored in JPEG format (.jpg) and are named using a consistent zero-padded numeric convention (e.g., 000,001.jpg, 000,002.jpg), which supports consistent file organization and straightforward file handling.

### 3.1 Repository structure

As summarized in Table 1, the root of *images/* contains seven main folders: 500, 1000, 2000, 5000, 10,000, 20,000, and Mix. Each denomination folder (500–20,000) contains images in which one or more banknotes of the same denomination appear within a frame. In contrast, the Mix folder contains images where multiple denominations may appear within a single frame; for images in the Mix folder, the dataset does not provide explicit per-image denomination listings, per-denomination presence indicators, or per-instance counts. As a result, these images are not directly usable for fully supervised multi-label training without additional annotation.

**Table 1:** “images” folder overview.

Folder name	Subfolder	Description of contents	File type
500	–	Images of 500 tenge banknotes	.jpg
1000	–	Images of 1000 tenge banknotes	.jpg
2000	–	Images of 2000 tenge banknotes	.jpg
5000	5000_new_single	Single new-design 5000 tenge banknote per image	.jpg
	5000_new	Multiple new-design 5000 tenge banknotes per image	.jpg
	5000_old	Multiple old-design 5000 tenge banknotes per image	.jpg
10,000	–	Images of 10,000 tenge banknotes	.jpg
20,000	–	Images of 20,000 tenge banknotes	.jpg
Mix	–	Images containing multiple denominations per image	.jpg

The 5000 folder is further subdivided to reflect both design series and scene composition, yielding three subfolders: 5000_new_single, 5000_new, and 5000_old (Table 1). This denomination is the only one subdivided in this manner because, at the time of data collection, the 5000 KZT banknote was actively circulating in both old and new designs, making design-based separation practically relevant for downstream recognition tasks. The 5000_new_single subset contains images with exactly one new-design 5000 KZT banknote per image, whereas 5000_new and 5000_old contain images with multiple banknotes per frame corresponding to the new and old design series, respectively. The single-banknote subset was additionally curated to address a common gap in prior resources: to the best of our knowledge, publicly available banknote datasets rarely provide a dedicated “single banknote per image” subset for the new-design 5000 KZT series, which can be useful for controlled denomination recognition and transfer-learning baselines.

All other denomination folders contain images with one or more banknotes of the same denomination per image and do not include further subfolder subdivisions. Labels correspond to folder names, and, except for the 5000 series, the dataset does not provide design-based subdivisions or instance counts per image.

**Fig. 1:**
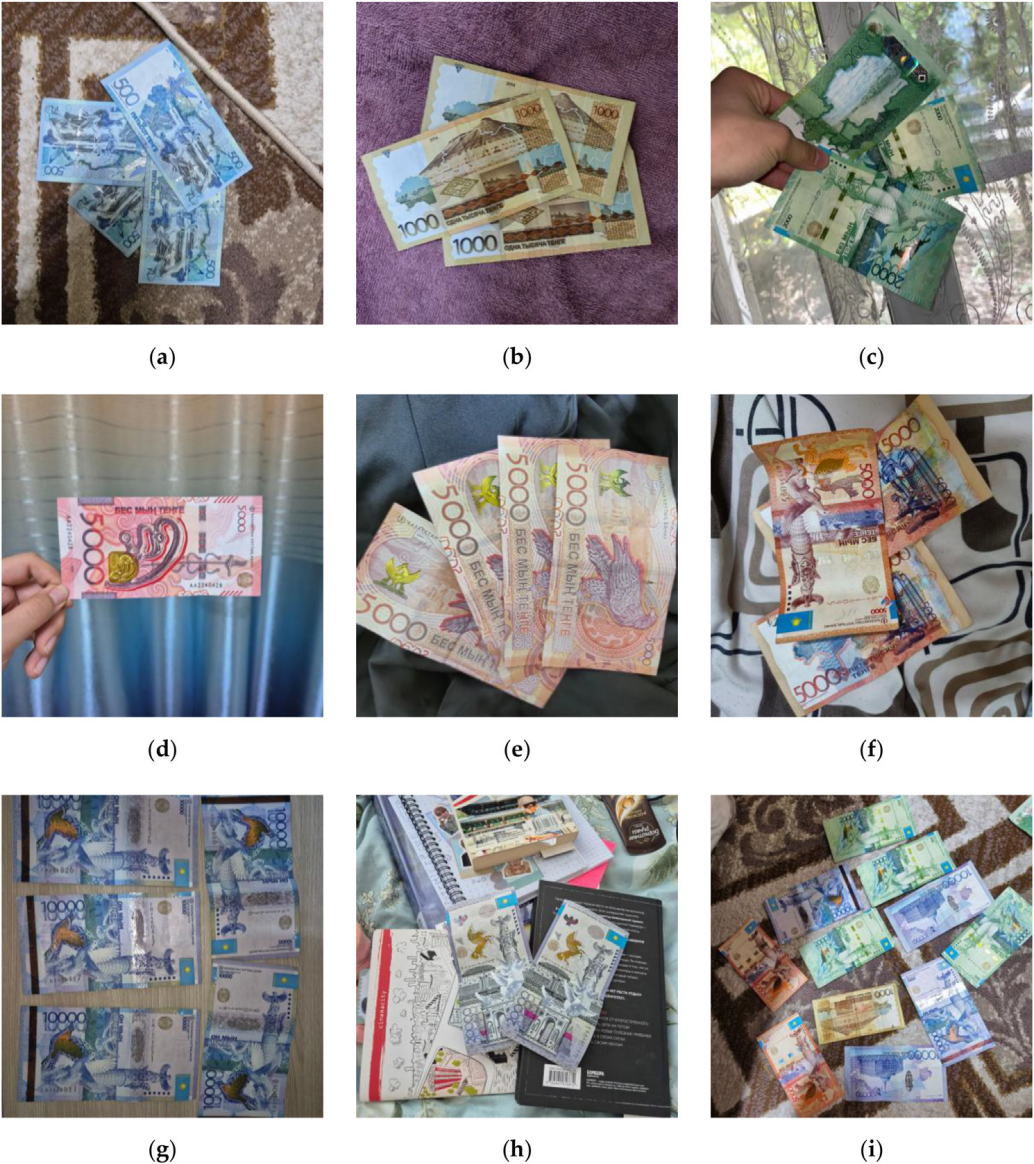
Representative sample images from each class folder: (a) for 500, (b) for 1000, (c) for 2000, (d) for 5000 new single, (e) for 5000 new, (f) for 5000 old, (g) for 10,000, (h) for 20,000, and (i) for Mix.

In Fig. 1, representative sample images from each class folder are presented to visually illustrate the typical appearance and capture variability in the dataset. The examples highlight differences in background context (e.g., carpet, fabric, wood surfaces, and everyday objects), illumination conditions (natural window light versus indoor ambient lighting), and viewpoints, including hand-held shots and partial occlusions. Fig. 1 also emphasizes variability in the number of banknotes per frame across classes. The “Mix” example (i) contains multiple denominations within a single image, reflecting the mixed-denomination setting used for multi-class scenarios.

In addition to the directory-based labels, the repository provides two auxiliary resources to support reproducibility and integrity checks. First, manifest.csv contains a per-file inventory with relative filepaths and SHA-256 checksums, allowing users to verify completeness and detect accidental file corruption. Hash matching can also be used to identify exact duplicates. Second, the *splits/* directory includes predefined *train/validation/test* split lists (*train.txt, val.txt*, and *test.txt*), where each file contains one relative image path per line (e.g., *images/<class>/<id>.jpg*). These split files enable consistent experimental partitioning without requiring users to re-sample the dataset. No pixel-level annotation files (e.g., bounding boxes or segmentation masks) are included; folder names and directory hierarchy remain the primary labeling mechanism for all images.

## 4 Experimental Design, Materials and Methods

This section documents (i) how the images were acquired, (ii) how they were organized and curated, and (iii) the software procedures that can be used to reproduce the dataset inventory and integrity checks.

### 4.1 Materials and targets

The dataset consists of photographs of Kazakhstani tenge banknotes collected for computer vision research. The captured categories include six single-denomination folders (500, 1000, 2000, 10,000, 20,000, and 5000) and one mixed-denomination folder (“Mix”), which contains frames where multiple denominations appear simultaneously. For the 5000 KZT denomination, images are further partitioned into three subsets reflecting both design series and scene composition: 5000_new_single (single banknote per image), 5000_new (multiple new-design banknotes per image), and 5000_old (multiple old-design banknotes per image).

### 4.2 Data acquisition checklist

Acquisition characteristics are summarized in Table 2. All images were captured using an Apple iPhone 15 smartphone camera. The built-in device camera and lens were used with default manufacturer settings; detailed lens metadata and flash usage statistics were not systematically logged. This reflects the dataset’s focus on uncontrolled, real-world acquisition conditions, rather than a fixed or laboratory-style capture protocol. Images were saved in JPEG (.jpg) format using the native iOS camera application. Image resolution varies across samples and reflects device-native output under different capture conditions, typically in the range produced by standard smartphone photography. Lighting conditions were intentionally varied and include natural daylight, indoor ambient lighting, and mixed illumination. Some images were captured with the device’s automatic flash enabled, while others were captured without flash; the exact proportion of flash versus non-flash images was not controlled or explicitly recorded. Backgrounds, capture distances, and viewing angles were not fixed and vary across images, reflecting everyday usage scenarios. No manual color correction, geometric correction, or post-processing was applied by the authors. Images retain the default iOS camera processing pipeline, including automatic exposure, white balance adjustment, tone mapping, and JPEG compression. Data collection took place in Almaty, Kazakhstan, between 10 Oct 2024 and 17 Nov 2024.

**Table 2:** Acquisition checklist.

Item	Description
Camera/device	Apple iPhone 15 smartphone camera
Lens	Built-in smartphone lens (manufacturer default; focal length metadata not recorded)
Image resolution	Varied; device-native JPEG resolutions from the iPhone 15, typically in the range of approximately 3000–4000 pixels on the longer image side.
File format	JPEG (.jpg)
Lighting	Varied lighting conditions including natural daylight and indoor ambient light. Some images were captured with the device’s automatic flash enabled, while others were captured without flash; the exact proportion of flash versus non-flash images was not recorded.
Backgrounds	Varied everyday backgrounds
Capture distance/angle	Varied
Collection period	10 Oct 2024 — 17 Nov 2024
Location	Almaty, Kazakhstan

### 4.3 Image capture protocol and scene design

Images were collected by photographing banknotes against a variety of backgrounds under varying illumination conditions. The dataset includes three main scene configurations:

• **Single-denomination scenes:** most denomination folders contain images with two or more banknotes of the same denomination within a frame.
• **Single-banknote subset (5000_new_single):** a controlled subset containing exactly one new-design 5000 KZT banknote per image.
• **Mixed-denomination scenes (Mix):** images where multiple denominations co-occur within the same frame.

This design allows researchers to study denomination recognition across both simpler single-content frames and more realistic cluttered frames with multiple objects and mixed content.

### 4.4 File naming and directory-based labeling

All images are stored in *JPEG (.jpg)* format and named using a fixed-width numeric convention (e.g., 000,001.jpg, 000,002.jpg). Labels are provided through the directory hierarchy: the top-level folder name corresponds to the denomination class, and for 5000 KZT the subfolder name additionally indicates design series and scene composition (single vs multiple banknotes).

### 4.5 Repository structure and contents

The repository is organized under an images/ directory with the following folders and subfolders:

• images/500/ → *.jpg
• images/1000/ → *.jpg
• images/2000/ → *.jpg
• images/5000/5000_new_single/ → *.jpg
• images/5000/5000_new/ → *.jpg
• images/5000/5000_old/ → *.jpg
• images/10,000/ → *.jpg
• images/20,000/ → *.jpg
• images/Mix/ → *.jpg

In addition to the images, the repository includes two reproducibility resources:

1. *manifest.csv* — a per-file inventory containing relative file paths and SHA-256 checksums. This file supports integrity verification (e.g., detecting missing files or accidental corruption) and enables reproducible dataset accounting.
2. *splits/* — predefined train/validation/test lists (train.txt, val.txt, test.txt), where each line corresponds to one image path relative to the repository root. These split files allow users to reproduce consistent experimental partitions without re-sampling the dataset.

### 4.6 Dataset inventory

The dataset contains 1846 JPEG images in total. Folder-level counts are reported in Table 3. The class distribution is intentionally not uniform, with the mixed-denomination folder containing a substantially larger number of images. This choice was made to increase the diversity and coverage of mixed-content scenes, providing more training examples for models intended to distinguish “mixed” frames from single-denomination frames and to learn robust features under clutter and co-occurrence.

**Table 3:** Image counts by folder/subfolder.

Path	Number of .jpg files
images/500/	100
images/1000/	112
images/2000/	100
images/5000/5000_new/	100
images/5000/5000_new_single/	100
images/5000/5000_old/	109
images/10,000/	110
images/20,000/	101
images/Mix/	1014

Because this imbalance can affect supervised learning, users may consider standard mitigation strategies depending on the task, such as (i) class-weighted losses, (ii) balanced sampling, (iii) reporting per-class metrics (macro-averaged F1/accuracy), or (iv) training with separate objectives (e.g., single-vs-mix screening followed by denomination-specific modeling). The provided fixed splits enable fair comparison of such strategies across studies.

The predefined train/validation/test splits preserve the original folder-level class proportions but were not generated using formal stratified sampling procedures. As a result, the inherent class imbalance—particularly the larger size of the Mix category—is maintained across all splits. Users requiring balanced or stratified training data may apply alternative resampling or reweighting strategies depending on their experimental goals.

### 4.7 Data curation and quality control

Curation was performed by organizing all images into denomination-specific folders, and specifically for the 5000 KZT denomination into design/scene subfolders that separate old versus new series and single-banknote versus multi-banknote compositions. This directory structure serves as the primary labeling mechanism and was applied consistently across the repository to ensure that each image can be traced to its intended class.

Quality control (QC) was conducted to improve dataset reliability and to minimize issues that commonly affect computer vision training pipelines. The following checks and cleaning steps were applied:

• *File format and readability validation:* All collected files were inspected to confirm they are readable JPEG images. Images that failed to open correctly or exhibited decoding errors were flagged and removed to prevent runtime failures during training or evaluation.
• *Folder-label consistency checks:* The directory-based labels were reviewed to ensure that images were placed in folders matching the intended denomination and, where applicable, the 5000 KZT design/scene category. This included verifying that the 5000 KZT subfolders reflect the correct series designation (new vs. old) and composition setting (single vs. multiple banknotes per frame), reducing the risk of label noise introduced by misplacement.
• *Removal of corrupted, duplicate, or near-duplicate files:* Corrupted images and exact duplicates were removed during curation. When visually or structurally near-duplicate frames were identified (e.g., highly similar shots captured in rapid succession with minimal variation), they were also excluded when appropriate to reduce redundancy and limit unintentional information leakage across potential train/validation/test splits.
• *Exclusion of low-quality or unsuitable content:* Images deemed unsuitable for robust model training—such as severely blurred frames, poorly exposed images that obscure key banknote details, or samples with excessive motion artifacts were removed. In addition, images containing personally identifiable content (e.g., recognizable faces) or other ethically inappropriate elements were excluded to improve compliance with common dataset ethics expectations and to ensure the dataset focuses strictly on banknote imagery.
• *Integrity and inventory support via cryptographic manifest:* To support reproducibility and integrity verification, a dataset inventory file (manifest.csv) was generated. The manifest stores a per-file record including relative file paths and SHA-256 checksums, enabling users to validate completeness after download, detect accidental corruption during transfer/storage, and confirm that files have not been unintentionally modified.

Together, these curation and QC steps aim to provide a clean, ethically safer, and technically reliable dataset that is straightforward to use in standard computer vision workflows while preserving realistic variability in capture conditions.

### 4.8 Reproducibility and integrity verification

The following minimal Python 3 scripts (i) generate the manifest, (ii) verify a local copy against the manifest by detecting missing files or checksum mismatches, and (iii) reproduce folder-level image counts from manifest.csv to support dataset reuse and to enable integrity checks after download or redistribution.

In the generated manifest, the series field functions as a lightweight categorical indicator. For denominations other than 5000 KZT, the value "current" is used as a neutral placeholder to indicate the absence of design-based subdivision and should not be interpreted as a formal design-series label.

**Script 1: Generate manifest (SHA-256)**

*import csv*

*import hashlib*

*from pathlib import Path*

def sha256_file(path: Path, chunk_size: int = 1024 * 1024) -> str:

*h = hashlib.sha256()*

*with path.open("rb") as f:*

*for chunk in iter(lambda: f.read(chunk_size), b""):*

*h.update(chunk)*

*return h.hexdigest()*

*root = Path("images")*

*out_path = Path("manifest.csv")*

*rows = []*

for img_path in sorted(root.rglob("*.jpg")):

*parts = img_path.parts # ("images", "<denom>", …, "<file>.jpg")*

*denomination = parts[* *1* *]*

*# series label*

*series = "current"*

*if denomination == "5000″ and len(parts) ≥ 3:*

*series = parts[* *2* *] # "5000_new", "5000_old", "5000_new_single"*

*elif denomination == "Mix":*

*series = "mixed"*

*rows.append({*

*"filepath": img_path.as_posix(),*

*"denomination": denomination,*

*"series": series,*

*"source": "original",*

*"sha256": sha256_file(img_path),*

*})*

*with out_path.open("w", newline="", encoding="utf-8") as f:*

*writer = csv.DictWriter(f, fieldnames=["filepath", "denomination", "series", "source", "sha256"])*

*writer.writeheader()*

*writer.writerows(rows)*

*print(f"Wrote {len(rows)} rows to {out_path}")*

**Script 2: Verify local files against manifest**

*import csv*

*import hashlib*

*from pathlib import Path*

def sha256_file(path: Path, chunk_size: int = 1024 * 1024) -> str:

*h = hashlib.sha256()*

*with path.open("rb") as f:*

*for chunk in iter(lambda: f.read(chunk_size), b""):*

*h.update(chunk)*

*return h.hexdigest()*

*manifest_path = Path("manifest.csv")*

*root = Path(".")*

*missing = []*

*mismatch = []*

*checked = 0*

*with manifest_path.open("r", encoding="utf-8") as f:*

*reader = csv.DictReader(f)*

*for row in reader:*

*rel = Path(row["filepath"])*

*expected = row["sha256"].strip().lower()*

*file_path = root / rel*

*if not file_path.exists():*

*missing.append(str(rel))*

*continue*

*actual = sha256_file(file_path).lower()*

*checked += 1*

*if actual != expected:*

*mismatch.append((str(rel), expected, actual))*

*print(f"Checked: {checked}")*

*print(f"Missing: {len(missing)}")*

*print(f"Checksum mismatches: {len(mismatch)}")*

**Script 3: Reproduce folder-level counts**

*import pandas as pd*

*df = pd.read_csv("manifest.csv")*

*df["top_folder"] = df["filepath"].str.split("/").str[1]*

*df["subfolder"] = df["filepath"].str.split("/").str[2].where(df["top_folder"] == "5000″, "-")*

*df.loc[df["top_folder"] != "5000″, "subfolder"] = "-"*

*counts = (df.groupby(["top_folder", "subfolder"])*

*.size()*

*.reset_index(name="n_images")*

*.sort_values(["top_folder", "subfolder"]))*

*print(counts.to_string(index=False))*

*print("Total images:", len(df))*

These scripts are intended as lightweight utilities for verifying dataset integrity and reproducing the reported inventory statistics.

## Limitations

The dataset is limited to images of Kazakhstani banknotes and therefore does not represent other currencies or cross-currency visual variations. As a result, its scope is restricted to denomination recognition and related tasks within a single national currency system. While each denomination folder contains a minimum number of images, the distribution of images across categories is not uniform, particularly due to the substantially larger size of the mixed-denomination category. This imbalance may require additional handling by users who rely on evenly distributed class sizes. The images were collected under varied real-world conditions, but they do not systematically cover all possible environments, camera devices, or extreme lighting scenarios. Certain viewpoints, occlusions, or degrees of physical wear may be underrepresented. In addition, backgrounds may lack certain challenging elements (e.g., reflective objects), which can limit robustness to glare or mirror-like surfaces in downstream applications. The dataset does not explicitly provide the count of banknotes per image in multi-banknote frames, which may constrain tasks that rely on precise instance counts. The dataset does not include pixel-level annotations such as bounding boxes or segmentation masks. Researchers interested in object detection or instance-level tasks must generate additional annotations if required. The dataset also does not include counterfeit banknote examples, so it is not directly suitable for anti-counterfeiting or authenticity verification studies. Although the dataset distinguishes between old and new designs of the 5000 tenge banknote, similar design-based subdivisions are not provided for other denominations.

## Ethics Statement

The authors confirm that they have read and complied with the ethical requirements for publication in Data in Brief. The current work does not involve human subjects, animal experiments, or data collected from social media platforms. All data consist exclusively of images of physical objects (banknotes) collected for research purposes and do not contain any personal, identifiable, or sensitive information.

## CRediT Author Statement

**Ualikhan Sadyk**: Conceptualization, Dataset curation, Methodology, Investigation, Writing – original draft, Visualization. **Makhambet Yerzhan**: Formal analysis, Software support, Data collection and acquisition, Dataset curation, Validation, Writing – review & editing. **Cemil Turan**: Supervision, Project administration, Writing – review & editing, Resources. **Haohan Wang**: Data processing workflow development, Technical support, Writing – review & editing, Funding acquisition.

## Data Availability

Mendeley DataKazakh Banknote Image Dataset (Original data)
